# Supplementary material for: Diagnostic Efficacy of Advanced Ultrasonography Imaging Techniques in Infants with Biliary Atresia (BA): A Systematic Review and Meta-Analysis
Source: Children (Basel). 2022 Oct 31;9(11):1676. doi: 10.3390/children9111676 (PMC9688715; doi:10.3390/children9111676)
Supplement: Supplementary file 1 [file children-09-01676-s001.zip › children-1958480-supplementary.pdf]

Database search strings

Database searched	Search strings
1. Pubmed	1. Biliary atresia AND ultrasonography AND (diagnostic accuracy)<br>2. Biliary atresia AND ultrasonography AND (diagnostic performance)
2. EBSCOhost (CINAHL and Medline)	1. TX biliary atresia AND TX ultrasonography AND TX diagnostic accuracy<br>2. TX biliary atresia AND TX ultrasonography AND TX diagnostic performance
3. Embase	1. Query('biliary atresia'/exp OR 'biliary atresia') AND ('ultrasonography'/exp OR ultrasonography) AND ('diagnostic accuracy'/exp OR 'diagnostic accuracy')<br>2. Query('bile duct atresia'/exp OR 'bile duct atresia'/exp/mj OR 'bile duct atresia') AND ('echography'/exp OR 'echography') AND ('diagnostic performance'/exp OR 'diagnostic performance')<br>3. Query('bile duct atresia'/exp OR 'bile duct atresia'/exp/mj OR 'bile duct atresia') AND ('echography'/exp OR 'echography') AND ('diagnostic accuracy'/exp OR 'diagnostic accuracy')
4. Web of Science Core Collection	((All=(biliary atresia)) AND ALL=(ultrasonography)) AND ALL=(diagnostic performance or diagnostic accuracy)
5. Google scholar	"biliary atresia" AND ultrasonography AND (diagnostic accuracy OR performance)
